# Epidemiology, risk profile, management, and outcome in geriatric patients with atrial fibrillation in two long-term care hospitals

**DOI:** 10.1038/s41598-022-22013-6

**Published:** 2022-11-04

**Authors:** Gernot Wagner, Michael Smeikal, Christoph Gisinger, Deddo Moertl, Stephan Nopp, Gerald Gartlehner, Ingrid Pabinger, Gerald Ohrenberger, Cihan Ay

**Affiliations:** 1 Department for Evidence-based Medicine and Evaluation, Danube University Krems, Krems, Austria; 2 Haus der Barmherzigkeit Tokiostraße, Vienna, Austria; 3 Haus der Barmherzigkeit Seeboeckgasse, Vienna, Austria; 4 Center for Geriatric Medicine and Geriatric Nursing, Danube University Krems, Krems, Austria; 5 Department of Internal Medicine 3, University Hospital St. Poelten, St. Poelten, Austria; 6 Karl Landsteiner University of Health Sciences, Krems, Austria; 7 Clinical Division of Haematology and Haemostaseology, Department of Medicine I, Medical University of Vienna, Vienna, Austria; 8 RTI International, Research Triangle Park, NC USA

**Keywords:** Atrial fibrillation, Epidemiology

## Abstract

Aim of this study was investigate the prevalence and incidence of atrial fibrillation (AF) and to describe the clinical characteristics, risk profiles, and types of anticoagulant therapy for stroke prevention and the clinical outcomes in persons admitted to a long-term care hospital. We conducted a retrospective cohort study using data from the electronic medical records of patients aged 65 years or older living in two long-term care hospitals between January 1, 2014 and October 31, 2017. Overall data from 1148 patients (mean age 84.1 ± 7.9 years, 74.2% women) were analyzed. At baseline, the median CHA_2_DS_2_-VASc score was 4 (IQR 3–5) and the HAS-BLED score 2 (IQR 2–3). We observed patients over a median period of 3.7 years. The point prevalence of AF was 29.6% (95% CI 25.8–33.7) on January 1, 2014. The 1-year cumulative incidence of de novo AF was 4.0% (2.8–5.6). Oral anticoagulants were prescribed in 48% of patients with AF. The cumulative incidence at 1 year for a composite outcome of TIA, stroke, or systemic arterial embolism was 0.6% (0.1–3.1) and 1.7% (0.5–4.6) and for bleeding 2.6% (0.9–6.2) and 1.8% (0.5–4.8) in patients with AF and oral anticoagulants or no oral anticoagulants, respectively. In long-term care hospital patients, we observed a high burden of AF. However, only about half of patients with AF received oral anticoagulation for stroke prevention.

## Introduction

Atrial fibrillation (AF) is the most common cardiac arrhythmia, and its incidence increases with age^1^. While the overall prevalence of AF in the general population is 1% to 2%^1–3^, it increases to around 4% in persons aged between 60 and 70 years and reaches almost 20% in those aged 85 years or older^4^. With life expectancy increasing, the proportion of geriatric patients aged 65 or older will constantly increase in the next decades^1^. This, in turn, will lead to a considerable increase in the proportion of patients with AF who are at an elevated risk of stroke, hospitalization, congestive heart failure, and death^5–8^.

Current guidelines recommend non-vitamin K antagonist oral anticoagulants (NOAC) in favor of vitamin K antagonists (VKA) for stroke prevention in patients with nonvalvular AF^9^. However, balancing the efficacy and safety of oral anticoagulation (OAC) in older adults with AF is challenging due to the increased risk of stroke and bleeding. The management of OAC in a geriatric population might further be complicated by frailty, polypharmacy, and impaired renal or liver function. These factors might contribute to the low prescription rates of OAC for stroke prevention in older adults with AF.

A high proportion of older persons unable to care for themselves live in long-term care facilities such as nursing homes and long-term care hospitals. Patients admitted to these institutions are usually of advanced age and suffer from various comorbidities, thus requiring permanent nursing and medical care. Although previous studies evaluated the prevalence of AF and the management of anticoagulation in older populations, the evidence on patients in nursing homes or long-term care hospitals is scarce^10,11^.

Therefore, the aim of this study was to gather epidemiologic data, such as the prevalence and incidence of AF, and describe the clinical characteristics, risk profiles, patterns of anticoagulant therapy, and the outcomes in older adults admitted to long-term care hospitals.

## Methods

### Study design

We conducted a retrospective cohort study using data from the electronic medical records of two long-term care hospitals. The Ethics Committee of the Medical University of Vienna approved this study (EK Nr. 2124/2017) and confirmed that no individual patient consent form is required for this retrospective data analysis. The study was conducted in accordance with relevant guidelines and regulations. For the reporting of this observational study, we followed the Strengthening the Reporting of Observational Studies in Epidemiology (STROBE) statement^12^.

### Study population and setting

We analyzed the consecutively collected data of older adults in two long-term care hospitals (Haus der Barmherzigkeit Tokiostrasse and Haus der Barmherzigkeit Seeboeckgasse, Vienna, Austria). These hospitals provide permanent nursing and specialized medical care for older adults, multimorbid and chronically ill patients with various levels of care dependency. We included patients aged 65 years or older, irrespective of the required care dependency level, who were already admitted on January 1, 2014 or were newly admitted thereafter, until October 31, 2017 (see Supplementary Fig. S1). The end of follow-up was October 31, 2017, resulting in a maximum follow-up of 3 years and 10 months.

### Data sources and variables

We obtained data on patient characteristics including clinical diagnosis and geriatric assessment, medication, laboratory results as well as date of admission and—if applicable—date of discharge from a long-term care hospital, information on discharge destination, and death. Based on the available data, we calculated the Charlson Comorbidity Index^13^, the CHA_2_DS_2_-VASc score (Congestive heart failure, Hypertension, Age ≥ 75 years, Diabetes mellitus, Stroke or transient ischemic attack [TIA], Vascular disease, Age 65 to 74 years, Sex category) for the risk of stroke^14^ and the HAS-BLED score (Hypertension, Abnormal Renal/Liver Function, Stroke, Bleeding History or Predisposition, Labile INR, Elderly, Drugs/Alcohol Concomitantly) score for the risk of bleeding^15^ at baseline. According to the European Society of Cardiology (ESC) guidelines for the diagnosis and management of AF, a high risk of bleeding was defined as a HAS-BLED score ≥ 3^9^.

For the baseline and outcome variables, we used a combination of diagnostic codes according to the International Classification of Disease, version 10 (ICD-10) assigned by treating physicians and/or free-text information from electronic medical records. In case the ICD-10 code for a certain diagnosis was missing, we relied on free-text information if unambiguously reported. The date of diagnosis was reconciled/verified with free-text information. For patients newly admitted after January 1, 2014, any diagnosis dated within 5 days after admission was considered past medical history. Time to diagnosis was calculated as the time from January 1, 2014 or admission to first documentation in the electronic medical record. Experienced clinicians from the study team clarified ambiguities related to disease diagnosis and dates. If necessary, electronic medical records were manually reviewed.

The Anatomical Therapeutic Chemical (ATC) classification was used to identify oral anticoagulants (VKA or NOAC comprising rivaroxaban, dabigatran, apixaban, edoxaban) and other medication of interest (parenteral anticoagulants, platelet inhibitors, antidiabetics, nonsteroidal anti-inflammatory drugs). We considered medication to be present at baseline if prescribed within 5 days after January 1, 2014 or if prescribed within 5 days after admission between January 2014 and October 2017.

### Outcome measures

The primary outcomes were the prevalence of AF and the proportion of patients with AF on OAC at baseline. The secondary outcomes were the incidence of de novo AF and a composite of TIA, stroke, or non-central nervous system (CNS) systemic arterial embolism as well as bleeding and all-cause death during follow-up. *AF* was defined as at least one diagnosis of paroxysmal, persistent, or permanent AF, typical or atypical atrial flutter, or unspecified AF and atrial flutter (ICD-10 code I48.X). *OAC* was defined as the prescription of oral anticoagulants (VKA or NOAC) documented in the medical records.

*TIA* was defined as a focal neurological deficit that fully resolved within 24 h (ICD-10 code G45.X). *Stroke* referred to any nontraumatic focal neurological deficit lasting at least 24 h, including any ischemic or hemorrhagic stroke (ICD-10 codes I61.X–64.X). *Non-CNS systemic arterial embolism* was defined as any noncerebral peripheral embolism leading to acute loss of blood flow to a peripheral artery (ICD-10 code I74.X). *Bleeding* included any clinically overt traumatic or nontraumatic intracranial, gastrointestinal, or other extracranial bleeding (e.g., ocular, skin and soft tissue, renal, retroperitoneal, pericardial, intra-articular). *Intracranial bleeding* included any epidural, subdural, subarachnoidal, or intracerebral bleeding. *Gastrointestinal bleeding* was defined as any clinically overt bleeding originating from the upper or lower gastrointestinal tract. For all outcome variables, we used a combination of diagnostic codes according to the ICD-10 code assigned by the treating physicians and/or free-text information from the electronic medical records as described above.

### Data management

We retrieved the patient data from the electronic medical records, following the current standards of patient data security. Authorized study personnel exported and pseudonymized the data from the central hospital management software to Microsoft Excel 2016 (Microsoft, Redmond, USA) spreadsheets in January 2018. We obtained cross-sectional data on January 1, 2014 and on admission thereafter. The end of follow-up was October 31, 2017. We checked eligible patients’ data for plausibility.

### Statistical analysis

For continuous baseline variables, we computed means and standard deviations if the data were approximately normally distributed; we calculated the median and the 25th and 75th percentiles for nonnormally distributed data. We visually assessed whether data are normal distributed. We reported categorical data as absolute and relative frequencies. On January 1 from 2014 to 2017, we estimated the prevalence and calculated a 95% confidence interval as the proportion of patients with AF (point prevalence). Patients without AF were compared to those with AF using two-sided t-tests or Wilcoxon tests in the case of a nonnormal distribution (continuous variables) and by chi-squared tests (categorical variables).

We calculated the time from baseline to death from any cause, discharge from long-term care hospital, or end of follow-up. Patients were censored at discharge from long-term care hospital or at the end of the follow-up period (October 31, 2017). A Kaplan–Meier analysis with 95% confidence intervals (CIs) was performed to estimate the mortality. We calculated the time to (1) de novo AF, (2) stroke, TIA, or systemic arterial embolism, (3) first occurrence of bleeding, or (4) death from any cause. If no events occurred, we calculated the time to discharge from long-term care hospital or the end of follow-up. For the clinical outcomes, we separately estimated the cumulative incidence for competing risks whereby death from any cause was taken as a competing risk (Fine & Gray). To identify possible risk factors for all-cause death, a composite of TIA, stroke, or systemic embolism, and bleeding in patients with AF, we calculated univariable competing risk regression models (cause-specific hazard model). If the p-value in a univariable model was less than 0.05, the respective variable entered a multivariable model. P-values less than 0.05 were considered statistically significant. SAS^®^ software version 9.4 (SAS Institute, Cary, NC, USA) and STATA release 14.1 (StataCorp LP, College Station, TX, USA) were used for the statistical analyses.

## Results

### Study population

**Figure 1:**
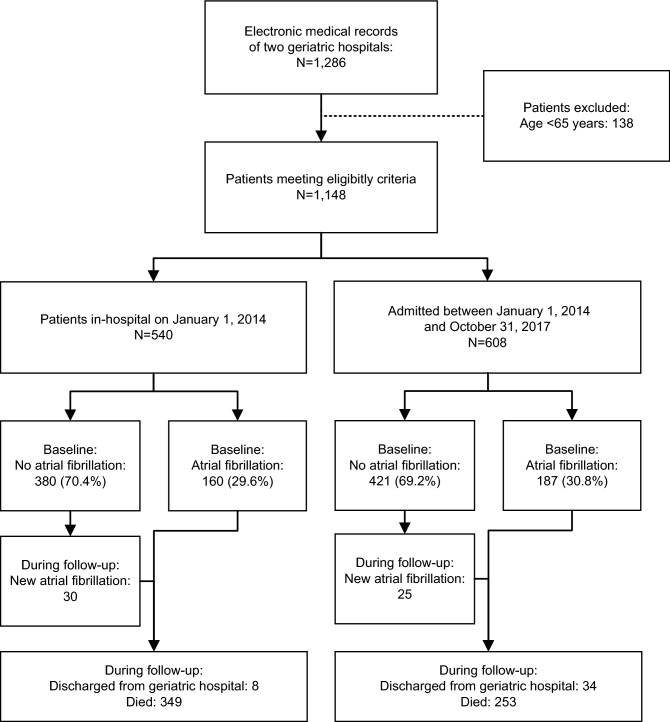
Flowchart of patient cohort. N, number of patients.

Overall, we analyzed data from 1148 patients of two long-term-care hospitals. The study flowchart (Fig. 1) shows the patient selection process and the number of patients in house on January 1, 2014 (N = 540) and newly admitted thereafter (N = 608) until October 31, 2017. The median follow-up was 3.7 years (interquartile range [IQR] 1.8–3.8). The mean age of the patients was 84.1 ± 7.9 years; 74.2% were female. We present the baseline characteristics of patients with AF (N = 347) and without AF (N = 801) in Table 1. Compared to patients without AF, patients with AF had more comorbidities such as hypertension, diabetes, heart failure, and ischemic and valvular heart disease as well as history of stroke or TIA. In AF patients with and without OAC, we found differences regarding body mass index (BMI) and HAS-BLED score as well as history of malignancy and bleeding (Supplementary Table S1). No patient received left atrial appendage occlusion or other forms of thromboembolic prophylaxis other than anticoagulation.

**Table 1:** Patients’ baseline characteristics.

Characteristic	All	No AF	AF	*P*
	N = 1148	N = 801	N = 347	
Age, years, mean ± SD	84.1 ± 7.9	83.6 ± 8.2	85.4 ± 7.0	< 0.001
Female, n (%)	852 (74.2)	597 (74.5)	255 (73.5)	0.71
BMI, kg/m^2^, mean ± SD *(105 missing)*	25.0 ± 5.7	24.9 ± 5.6	25.2 ± 6.0	0.50
**Charlson Comorbidity Index, median [IQR]**	2 [1–3]	2 [1–3]	2 [1–4]	< 0.001
0–1, n (%)	443 (38.6)	354 (44.2)	89 (25.7)	
2–3, n (%)	512 (44.6)	342 (42.7)	170 (49.0)	
≥ 4, n (%)	193 (16.8)	105 (13.1)	88 (25.4)	
**CHA**_**2**_**DS**_**2**_**-VASc score, median [IQR]**	4 [3–5]	4 [3–5]	5 [4–6]	< 0.001
0–1, n (%)	19 (1.7)	18 (2.3)	1 (0.3)	
2–3, n (%)	281 (24.5)	240 (30.0)	41 (11.8)	
4–5, n (%)	569 (49.6)	403 (50.3)	166 (47.8)	
≥ 6, n (%)	279 (24.3)	140 (17.5)	139 (40.1)	
**HAS-BLED score, median [IQR]**	2 [2–3]	2 [2–3]	2 [2–3]	0.005
0–2, n (%)	657 (57.2)	473 (59.1)	184 (53.0)	
≥ 3, n (%)	491 (42.8)	328 (41.0)	163 (47.0)	
High care dependency, n (%) *(50 missing)*	725 (66.0)	510 (66.3)	215 (65.4)	0.76
Increased risk of falling, n (%) *(47 missing)*	780 (70.8)	552 (71.5)	228 (69.3)	0.46
**Medical history, n (%)**				
Hypertension	777 (67.7)	514 (64.2)	263 (75.8)	< 0.001
Hyperlipidemia	243 (21.2)	173 (21.6)	70 (20.2)	0.59
Diabetes mellitus	312 (27.2)	197 (24.6)	115 (33.1)	< 0.01
Heart failure/cardiomyopathy	202 (17.6)	82 (10.2)	120 (34.6)	< 0.001
Ischemic heart disease	288 (25.1)	172 (21.5)	116 (33.4)	< 0.001
Valvular heart disease	113 (9.8)	47 (5.9)	66 (19.0)	< 0.001
Mitral stenosis	4 (0.4)	1 (0.1)	3 (0.9)	0.09
Mechanical heart valve	2 (0.6)	0	2 (0.6)	0.09
Previous stroke or TIA	316 (27.5)	174 (21.7)	142 (40.9)	< 0.001
Peripheral artery disease	91 (7.9)	52 (6.5)	39 (11.2)	< 0.01
Chronic renal insufficiency	266 (23.2)	161 (20.1)	105 (30.3)	< 0.001
Solid or hematologic malignancy ^a^	167 (14.6)	116 (14.5)	51 (14.7)	0.92
Dementia	649 (56.5)	470 (58.7)	179 (51.6)	0.03
Bleeding	126 (11.0)	85 (10.6)	41 (11.8)	0.55
**Oral anticoagulants**^**b**^**, n (%)**				
Any	195 (17.0)	27 (3.4)	168 (48.4)	< 0.001
VKA	61 (5.3)	7 (0.9)	54 (15.6)	< 0.001
NOAC	134 (11.7)	20 (2.5)	114 (32.9)	< 0.001
Apixaban	14 (1.2)	1 (0.1)	13 (3.8)	< 0.001
2.5 mg	10 (0.9)	1 (0.1)	9 (2.6)	
5.0 mg	4 (0.4)	0	4 (1.2)	
Rivaroxaban	100 (8.7)	15 (1.9)	85 (24.5)	< 0.001
10 mg	5 (0.4)	1 (0.1)	4 (1.2)	
15 mg	58 (5.1)	8 (1.0)	50 (14.4)	
20 mg	37 (3.2)	6 (0.8)	31 8.9)	
Dabigatran	13 (1.1)	3 (0.4)	10 (2.9)	< 0.001
75 mg	1 (0.1)	0	1 (0.3)	
110 mg	9 (0.8)	3 (0.4)	6 (1.7)	
150 mg	3 (0.3)	0	3 (0.9)	
Edoxaban	7 (0.6)	1 (0.1)	6 (1.7)	< 0.01
30 mg	4 (0.4)	1 (0.1)	3 (0.9)	
60 mg	3 (0.3)	0	3 (0.9)	
**Parenteral anticoagulants**^**b**^**, n (%)**				
LMWH	181 (15.8)	105 (13.1)	76 (21.9)	< 0.001
Fondaparinux	2 (0.2)	2 (0.3)	0	1.00
**Platelet inhibitors**^**b**^**, n (%)**				
Any	304 (26.5)	251 (31.3)	53 (15.3)	< 0.001
ASS	247 (21.5)	204 (25.5)	43 (12.4)	< 0.001
Clopidogrel	70 (6.1)	58 (7.2)	12 (3.5)	0.01
Prasugrel	1 (0.1)	0	1 (0.3)	0.30
Ticagrelor	1 (0.1)	1 (0.1)	0	1.00
Dual antiplatelet therapy	15 (1.3)	12 (1.5)	3 (0.9)	0.57

AF, atrial fibrillation; ASS, acetylsalicylic acid; BMI, body mass index; IQR, interquartile range; LMWH, low-molecular-weight heparin; n, number of patients; NOAC, non-vitamin K antagonist oral anticoagulant; *P*, p-value; SD, standard deviation; TIA, transient ischemic attack; VKA, vitamin K antagonists.

^a^Excluding nonmelanoma skin cancer. Patients with multiple malignancies were counted once.

^b^Prescribed within 5 days after January 1, 2014 or after admission between January 2014 and October 2017.

### Prevalence of AF, risk profile, and anticoagulant therapy

Prevalences on January 1 of the respective year ranged from 29.6 to 32.5% in the years from 2014 to 2017 (Table 2). Among 608 patients newly admitted during the study period, 187 had AF, which translated to a period prevalence of 30.8% (95% CI 27.1–34.6). All patients with AF had CHA_2_DS_2_-VASc scores of 2 or greater, except one male patient with a score of 1. Oral anticoagulants were prescribed in 48.4% (168 of 347) of patients with AF.

**Table 2:** Point prevalences of AF from year 2014 to 2017 on January 1.

	January 1, 2014	January 1, 2015	January 1, 2016	January 1, 2017
Number of patients	540	535	526	516
Number of patients with AF	160	159	171	163
Proportion of patients with AF (95% CI)	29.6% (25.8–33.7)	29.7% (25.9–33.8)	32.5% (28.5–36.7)	31.6% (27.6–35.8)

AF, atrial fibrillation; CI, confidence interval.

### Incidence of de novo AF

During the study period, de novo AF occurred in 55 of 801 (6.9%) patients with no AF at baseline. The cumulative 1-, 2-, and 3-year incidences of de novo AF are presented in Table 3.

**Table 3:** Number of events and cumulative incidence during follow-up.

Outcome	No AF		AF with OAC		AF without OAC		*P*^c^
	N = 801		N = 168		N = 179		
	No. of events	Cumulative incidence (95% CI)	No. of events	Cumulative incidence (95% CI)	No. of events	Cumulative incidence (95% CI)	
**De novo AF**	55		–	–	–	–	
1 year	30	4.0 (2.8–5.6)	–	–	–	–	
2 years	47	6.7 (5.0–8.8)	–	–	–	–	
3 years	50	7.3 (5.5–9.4)	–	–	–	–	
**TIA, stroke, or systemic embolism**	27		6		9		0.26
1 year	16	2.2 (1.3–3.4)	1	0.6 (0.1–3.1)	3	1.7 (0.5–4.6)	
2 years	22	3.1 (2.0–4.6)	5	3.6 (1.3–7.7)	6	3.7 (1.5–7.5)	
3 years	25	3.7 (2.4–5.3)	5	3.6 (1.3–7.7)	7	4.5 (2.0–8.6)	
**Bleeding**^**a**^	22		8		5		0.64
1 year	17	2.3 (1.4–3.6)	4	2.6 (0.9–6.2)	3	1.8 (0.5–4.8)	
2 years	19	2.6 (1.6–4.0)	5	3.4 (1.3–7.4)	3	1.8 (0.5–4.8)	
3 years	21	3.0 (1.9–4.5)	6	4.3 (1.7–8.6)	4	2.6 (0.8–6.3)	
**All-cause death**^**b**^	406		82		114		< 0.001
1 year	195	26.2 (23.1–29.5)	33	21.3 (15.6–28.6)	71	41.5 (34.5–49.4)	
2 years	292	41.8 (38.1–45.6)	54	37.5 (30.0–46.1)	92	56.0 (48.4–63.8)	
3 years	363	55.1 (51.2–59.1)	73	54.6 (46.1–63.6)	103	64.9 (57.1–72.6)	

AF, atrial fibrillation; CI, confidence interval; N, number of patients; No., number; OAC, oral anticoagulation; TIA, transient ischemic attack.

^a^Intracranial bleeding, gastrointestinal bleeding, or other clinical relevant extracranial bleeding.

^b^1-Kaplan–Meier estimate.

^c^Univariable competing risk regression model (cause-specific hazard model); AF with OAC versus AF without OAC.

### Clinical outcomes

#### Incidence of stroke, TIA, or systemic arterial embolism

A composite endpoint of stroke, TIA, or systemic arterial embolism occurred in 42 of 1148 patients (3.7%). We observed 32 strokes, 9 TIAs, and 3 systemic arterial embolisms. In the competing risk analysis, the cumulative incidence of stroke, TIA, or systemic arterial embolism in patients suffering from AF who received OAC increased from 0.6 to 3.6% from year 1 to year 3. Comparatively, in patients with AF who were not treated with OAC, the cumulative incidence increased from 1.7 to 4.5% (see Table 3). No significant differences in the risk of stroke, TIA, or systemic arterial embolism were found in AF patients with and without OAC (see Table S2).

#### Incidence of bleeding

Thirty-six bleeding events (15 intracranial, 12 gastrointestinal, and 9 other extracranial bleedings) occurred in 35 patients. In the competing risk analysis, the 1-, 2-, and 3-year cumulative incidences of bleeding were numerically lower in AF patients without OAC compared to those with OAC (see Table 3). No significant difference in the risk of bleeding was found in AF patients with and without OAC (see Table S2).

We provide a cumulative incidence curve for the composite endpoint of stroke, TIA, systemic embolism or bleeding according to AF and OAC in the supplementary material (Supplementary Fig. S2).

#### Risk of all-cause death

**Figure 2:**
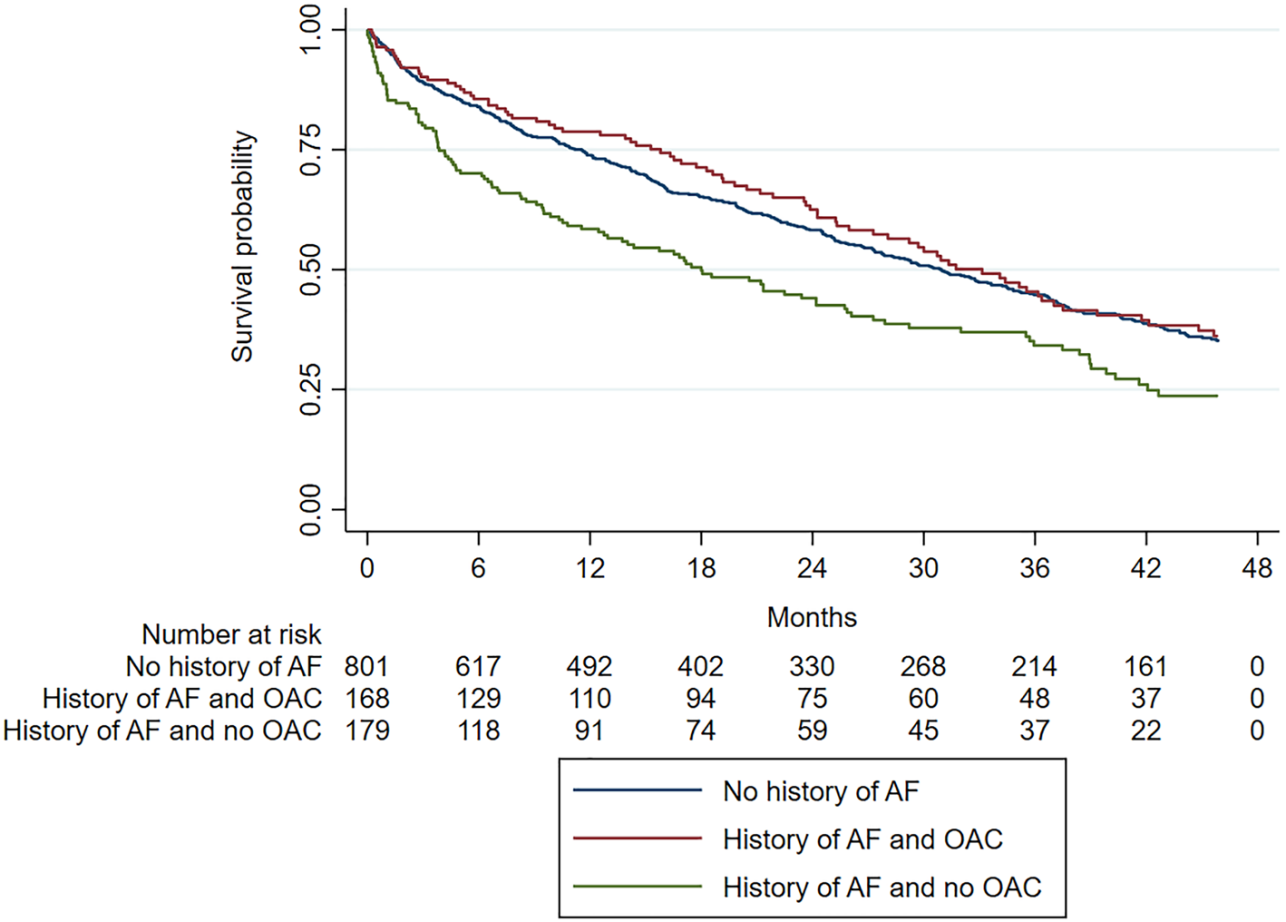
Death from any cause in patients with and without history of AF. AF, atrial fibrillation; OAC, oral anticoagulation.

Of the 602 patients (52.4%) who died during the study period, 196 had AF and 406 had no AF at baseline. The Kaplan–Meier analyses revealed lower 1-, 2-, and 3-year all-cause mortality in AF patients with OAC than in those without (Fig. 2). In patients with AF, OAC was associated with a significantly lower risk of death in a univariate analysis (HR 0.62; 95% CI 0.47–0.82, p < 0.001), but was no independent predictor of death in a multivariable regression analysis including age, female gender, and BMI (HR 0.77; 95% CI 0.56–1.06, p = 0.11; see Table S2).

## Discussion

In this retrospective cohort study in older adults admitted to long-term care hospitals, we found a high prevalence of AF. Despite a CHA_2_DS_2_-VASc score ≥ 2 in 99% and ≥ 4 in 88% of patients with AF, only 48% received OAC.

In our study, the point prevalence of AF ranged from 29.6 to 32.5%. This is higher compared to the prevalences reported in previous observational studies. A cross-sectional study from France found AF in 10.1% of nursing home residents^10^. Similarly, the US National Nursing Home Survey reported a prevalence of 10.9% in the year 2004^16^. Tulner et al. observed a prevalence of 20.1% in geriatric outpatients visiting a day clinic^11^. These differences from our findings may be explained by variations in the study populations and settings. Although the mean age of the patients in our study was similar, they suffered from various preexisting conditions and risk factors. The patients in this study were admitted to a long-term care hospital that offers their patients various health care facilities and permanent medical care to a greater extent than nursing homes. Therefore, these patients tend to be sicker with higher AF rates than patients in nonmedical nursing homes. In addition, regular medical check-ups in long-term care hospitals might lead to a higher AF detection rate.

Although all except one patient with AF had a class IA recommendation for OAC^17^, only half received it. In comparison, a smaller proportion of patients received low-molecular-weight heparin (16%) and platelet inhibitors (27%). Although these are nonstandard therapies for stroke prevention in patients with AF, patients might have received those treatments for indications other than stroke prevention. Previous studies also observed a high proportion of patients with AF who did not receive OAC despite an increased risk of stroke^18^. A large cross-sectional study from the USA found that 47.8% of nursing home residents with AF received OAC^19^. Similarly, Bahri et al. reported that a proportion of 49.1% of nursing home residents with AF in France received anticoagulants^10^. A systematic review found variable proportions of long-term care residents with AF receiving warfarin ranging from 17 to 57%^20^.

According to a French survey among physicians caring for nursing home residents, the main reasons for not prescribing anticoagulants were recurrent falls, cognitive impairment, and advanced age^10^. The patient characteristics of this study seem to be comparable to our study regarding age and CHA_2_DS_2_-VASc score. Therefore, these factors might also have been reasons for not prescribing OAC in patients with AF in our cohort study. Further, the risk of bleeding and prognosis of underlying conditions not reflected by established risk prediction tools might have influenced the decision on OAC. Although 47% of patients in our study had an HAS-BLED score ≥ 3, the bleeding risk in AF patients with OAC did not seem higher than in patients without.

Despite a high CHA_2_DS_2_-VASc score, we observed a low incidence rate of TIA, stroke, or systemic embolism events among patients with AF and OAC (1.9 per 100 person-years) and no OAC (3.5 per 100 person-years). An analysis of a German health claim database observed similar rate of stroke or systemic embolism of 1.8 per 100 person-years in patients aged 75 or older receiving OAC due to AF who had a similar mean CHA_2_DS_2_-VASc score^21^. However, outcome definitions were different. The low rate of TIA, stroke, or systemic embolism events in patients without OAC in our cohort study might be explained by the administration of low-molecular-weight heparin. Due to impaired renal function, even a prophylactic dose might have been sufficient for therapeutic anticoagulation. Another explanation for the low rate of stroke and systemic embolism during follow-up in our study population could have been the optimized management of other modifiable risk factors and comorbidities not represented in the CHA_2_DS_2_-VASc score.

Despite the high prevalence of AF at baseline we observed a high incidence of AF during follow-up. This was most probably facilitated by a high detection rate due to the high level of medical attention in our study, implying that physicians should not refrain from long-term surveillance of AF in this setting.

OAC was associated with a lower mortality in patients with AF, compared to patients with AF and without OAC. Despite that OAC has demonstrated its potential to reduce mortality^22^, this benefit is usually driven by a reduction of thromboembolic events. In our study OAC was an independent predictor of mortality; however as soon as other prognostic factors were included in the multivariable analysis, OAC was not associated with a difference in mortality. Therefore, the observed mortality reduction in our geriatric population is most probably not caused by OAC itself but can be explained by measured and unmeasured confounders. Patient characteristics associated with a higher morbidity and mortality might have contributed to the avoidance of OAC in certain patients with AF.

Our study has several limitations. First, we conducted a retrospective study based on electronic medical records with an additional selective manual medical record review susceptible to different types of bias. We might have missed or misclassified relevant information related to baseline and outcome diagnoses. We could not classify bleedings by common bleeding definitions, e.g. according to the Bleeding Academic Research Consortium (BARC) and the International Society on Thrombosis and Haemostasis (ISTH), because not all bleeding components were routinely collected. However, we considered that most bleedings mentioned in the medical records had a clinical significance sufficient to be classified as major bleedings. This must be considered, when comparing our bleeding rates to reports from other studies. Second, the study population included patients from two long-term care geriatric institutions. Therefore, the findings from this study cannot be extrapolated to other geriatric settings such as community, nursing homes, or acute care hospitals. Third, we were not able to obtain additional information why physicians decided for or against OAC or for the use of OAC with reduced dose based on individual patient characteristics or preferences. Therefore, we were not able to explore the possible confounding effect of factors that contributed to the physician’s decision to either not prescribe OAC at all or prescribe it in a reduced doses, or whether the decision was justified or regularly reviewed. Forth, patients with AF receiving low-molecular-weight heparin instead of OAC might confound our results regarding clinical outcomes. Finally, results from regression analysis of this cohort study should be interpretated with caution. Due to the low number of stroke, TIA, systemic arterial embolism, and bleeding events, we might have missed a significant influence of OAC on these events in our analyses.

In long-term care hospital patients, we observed a high prevalence of AF. However, only approximately half of patients with AF received OAC for stroke prevention despite a clear indication. To understand the underlying reasons for our findings, large prospective multicenter studies in patients admitted to long-term care hospitals are required. This might help further optimize and individualize stroke and bleeding prevention in older adults with AF.

## Supplementary Information

Supplementary Information.

## Data Availability

The dataset analyzed during the current study is available from the corresponding author on reasonable request.
