# Supplementary material for: Epidemiology, risk profile, management, and outcome in geriatric patients with atrial fibrillation in two long-term care hospitals
Source: Sci Rep. 2022 Nov 4;12:18725. doi: 10.1038/s41598-022-22013-6 (PMC9636160; doi:10.1038/s41598-022-22013-6)
Supplement: Supplementary file 1 — Supplementary Information. [file 41598_2022_22013_MOESM1_ESM.docx]

**Epidemiology, risk profile, management, and outcome in geriatric patients with atrial fibrillation in two long-term care hospitals**

Gernot Wagner, Michael Smeikal, Christoph Gisinger*,* Deddo Moertl, Stephan Nopp, Gerald Gartlehner, Ingrid Pabinger, Gerald Ohrenberger, Cihan Ay

# Supplementary Material

## Supplementary Table S1. Baseline characteristics of AF patients with and without OAC

| **Characteristic** | **No OAC** | **OAC** |  |
| --- | --- | --- | --- |
|  | **N=179** | **N=168** | ***P*** |
| Age, years, mean ± SD | 85.9 ± 7.0 | 84.8 ± 6.9 | 0.12 |
| Female, n (%) | 129 (72.1) | 126 (75.0) | 0.54 |
| BMI, kg/m^2^, mean ± SD *(36 missing)* | 23.9 ± 5.6 | 26.6 ± 6.1 | <0.001 |
| Charlson Comorbidity Index, median [IQR] | 2 [1–4] | 2 [1–3] | 0.54 |
| 0–1, n (%) | 46 (25.7) | 43 (25.6) |  |
| 2–3, n (%) | 83 (46.4) | 87 (51.8) |  |
| ≥4, n (%) | 50 (27.9) | 38 (22.6) |  |
| CHA_2_DS_2_-VASc score, median [IQR] | 5 [4–6] | 5 [4–6] | 0.15 |
| 0–1, n (%) | 0 | 1 (0.6) |  |
| ≥2, n (%) | 179 (100.0) | 167 (99.4) |  |
| HAS-BLED score, median [IQR] | 3 [2–3] | 2 [2–3] | <0.01 |
| 0–2, n (%) | 84 (46.9) | 100 (59.5) |  |
| ≥3, n (%) | 95 (53.1) | 68 (40.5) |  |
| High care dependency, n (%) *(18 missing)* | 110 (66.7) | 105 (64.0) | 0.61 |
| Increased risk of falling, n (%) *(18 missing)* | 113 (68.1) | 115 (70.6) | 0.63 |
| **Medical history, n (%)** |  |  |  |
| Hypertension | 132 (73.7) | 131 (78.0) | 0.36 |
| Hyperlipidemia | 30 (16.8) | 40 (23.8) | 0.10 |
| Diabetes mellitus | 53 (29.6) | 62 (36.9) | 0.15 |
| Heart failure/Cardiomyopathy | 58 (32.4) | 62 (36.9) | 0.38 |
| Ischemic heart disease | 58 (32.4) | 58 (34.5) | 0.68 |
| Valvular heart disease | 29 (16.2) | 37 (22.0) | 0.17 |
| Mitral stenosis | 3 (1.7) | 0 | 0.25 |
| Mechanical heart valve | 0 | 2 (1.2) | 0.23 |
| Previous stroke or TIA | 71 (39.7) | 71 (42.3) | 0.62 |
| Peripheral artery disease | 19 (10.6) | 20 (11.9) | 0.70 |
| Chronic renal insufficiency | 49 (27.4) | 56 (33.3) | 0.23 |
| Solid or hematologic malignancy ^a^ | 34 (19.0) | 17 (10.1) | 0.02 |
| Dementia | 88 (49.2) | 91 (54.2) | 0.35 |
| Bleeding | 30 (16.8) | 11 (6.6) | <0.01 |
| **Oral anticoagulants^b^, n (%)** |  |  |  |
| Any | 0 | 168 (100) | <0.001 |
| VKA | 0 | 54 (32.1) | <0.001 |
| NOAC | 0 | 114 (67.9) | <0.001 |
| **Parenteral anticoagulants^b^, n (%)** |  |  |  |
| LMWH | 73 (40.8) | 3 (1.8) | <0.001 |
| Fondaparinux | 0 | 0 |  |
| **Platelet inhibitors^b^, n (%)** |  |  |  |
| Any | 46 (25.7) | 7 (4.2) | <0.001 |
| ASS | 37 (20.7) | 6 (3.6) | <0.001 |
| Clopidogrel | 9 (5.0) | 3 (1.8) | 0.10 |
| Prasugrel | 1 (0.6) | 0 | 1.00 |
| Ticagrelor | 0 | 0 |  |
| Dual antiplatelet therapy | 1 (0.6) | 2 (1.2) | 0.61 |

**Abbreviations:** AF, atrial fibrillation; ASS, acetylsalicylic acid; BMI, body mass index; IQR, interquartile range; LMWH, low-molecular-weight heparin; n, number of patients; NOAC, non-vitamin K antagonist oral anticoagulant; OAC, oral anticoagulation; SD, standard deviation; TIA, transient ischemic attack; VKA, vitamin K antagonists

^a^ Excluding nonmelanoma skin cancer. Patients with multiple malignancies were counted once.

^b^ Prescribed within 5 days after January 1, 2014 or after admission between January 2014 and October 2017

## Supplementary Table S2. Predictors of clinical outcomes in patients with AF (N=347)

|  | **Univariable** |  |  | **Multivariable**  **adjusted** |  |
| --- | --- | --- | --- | --- | --- |
|  | **HR (95% CI)** | ***P*** |  | **HR (95% CI)** | ***P*** |
| **TIA, stroke, or systemic embolism** |  |  |  |  |  |
| Age | 0.98 (0.92–1.06) | 0.64 |  |  |  |
| Female | 1.00 (0.28–3.58) | 0.99 |  |  |  |
| OAC | 0.55 (0.20–1.55) | 0.26 |  |  |  |
| Diabetes | 1.15 (0.39–3.35) | 0.80 |  |  |  |
| Hypertension | 4.76 (0.63–36.27) | 0.13 |  |  |  |
| History of TIA or stroke | 1.58 (0.57–4.35) | 0.38 |  |  |  |
|  |  |  |  |  |  |
| **Bleeding** |  |  |  |  |  |
| Age | 1.00 (0.92–1.08) | 0.92 |  |  |  |
| Female | 1.36 (0.30–6.15) | 0.69 |  |  |  |
| OAC | 1.30 (0.43–4.00) | 0.64 |  |  |  |
| Diabetes | 0.41 (0.09–1.84) | 0.24 |  |  |  |
| Hypertension | 4.20 (0.55–32.37) | 0.17 |  |  |  |
| History of TIA or stroke | 0.57 (0.18–1.86) | 0.36 |  |  |  |
|  |  |  |  |  |  |
| **Death from any cause** |  |  |  |  |  |
| Age | 1.03 (1.01–1.06) | <0.01 |  | 1.04 (1.01–1.06) | <0.01 |
| Female | 0.58 (0.43–0.78) | <0.001 |  | 0.53 (0.38–0.76) | <0.001 |
| OAC | 0.62 (0.47–0.82) | <0.001 |  | 0.77 (0.56–1.06) | 0.11 |
| Diabetes | 1.25 (0.94–1.68) | 0.13 |  |  |  |
| Hypertension | 0.97 (0.70–1.34) | 0.83 |  |  |  |
| History of TIA or stroke | 1.06 (0.80–1.41) | 0.67 |  |  |  |
| BMI | 0.95 (0.92–0.98) | <0.001 |  | 0.96 (0.93–0.99) | <0.01 |
| Solid or hematologic malignancy | 1.76 (1.23–2.52) | <0.01 |  | 1.40 (0.93–2.12) | 0.11 |
| History of bleeding | 0.75 (0.48–1.18) | 0.22 |  |  |  |
| HAS-BLED score | 1.01 (0.88–1.17) | 0.86 |  |  |  |

**Abbreviations**: AF, atrial fibrillation; BMI, body mass index; CI, confidence interval; HR, hazard ratio; N, number of patients; OAC, oral anticoagulation; TIA, transient ischemic attack

## Supplementary Figure S1. Illustration of the study design

## Supplementary Figure S2. Cumulative incidence curve for the composite endpoint of stroke, TIA, systemic embolism, or bleeding according to atrial fibrillation and oral anticoagulation


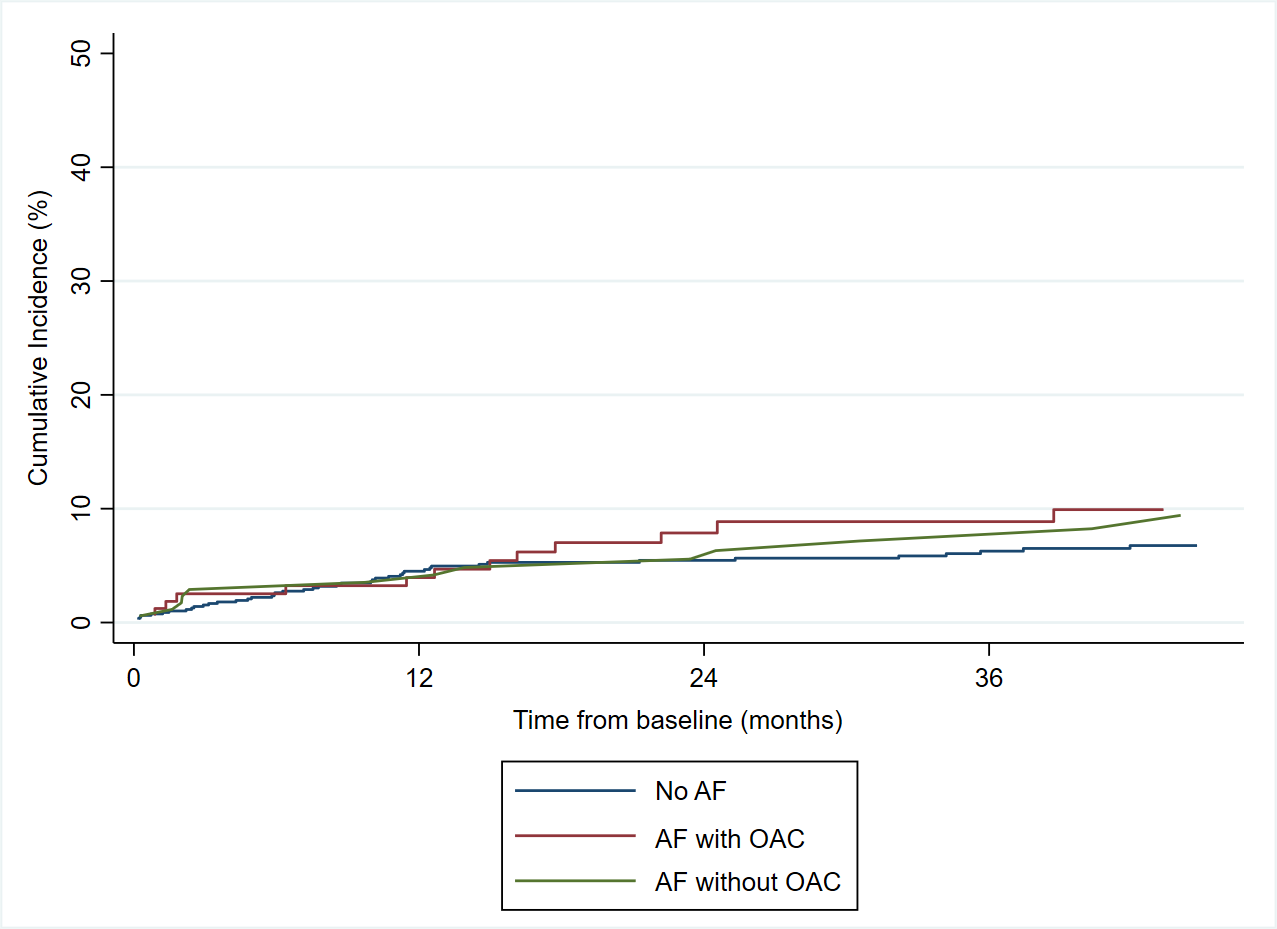


Note: The y-axis scale is limited to 50%.
